# Dataset concerning plasmonic thermal destruction of murine melanoma by gold nanoparticles obtained by green chemistry

**DOI:** 10.1016/j.dib.2020.105370

**Published:** 2020-02-29

**Authors:** Sabrina Pesnel, Yang Zhang, Fu Weiling, Anne-Laure Morel

**Affiliations:** a TORSKAL Nanosciences, 2 Rue Maxime Rivière, 97490, Sainte Clotilde, France; b Southwest Hospital, Third Military Medical University (TMMU), Chongqing, China

**Keywords:** Photothermal therapy, Gold nanoparticles, Murine melanoma, Cancer treatment, Green chemistry

## Abstract

The data presented in this article are related to the research paper “Proof of concept of plasmonic thermal destruction of surface cancers by gold nanoparticles obtained by green chemistry” (Ben Hadadda et *al*, 2019). In this article, we examined plasmonic thermal destruction of murine melanoma using gold nanoparticles obtained by green chemistry. The presented data were obtained by measuring tumor volume and mice weight in different groups of mice murine melanoma B16F10 treated or not with the nanoparticles and coupled to laser irradiation. These data were compared to the clinical reference treatment: anti-PD1 monoclonal antibody. The data were analyzed in order to be able to compare the antitumor effect of our treatment (photothermal plasmonic therapy using gold nanoparticles) and the reference treatment.

**Table:** Specifications Table

Subject	ONCOLOGY, NANOTECHNOLOGY
Specific subject area	Cancer, gold nanoparticles, photothermal therapy
Type of data	Table Figure
How data were acquired	Weight: the animals were weighed with a scale Tumor volume: The tumor dimensions (width, length, thickness) were measured with a digital caliper. This formula was used to calculate the volume: volume = (width x length x thickness)/2
Data format	Raw data & Analyzed
Parameters for data collection	The raw data were obtained from 25 melanoma bearing mice divided into 5 groups: - Group 1: injection of nanoparticles without laser - Group 2: Laser exposure alone - Group 3: Nanoparticles combined with laser - Group 4: Reference treatment group (anti-PD1 monoclonal antibody). Throughout this experimentation, mice were weighed and their clinical state was rigorously controlled.
Description of data collection	The experimentation started when the tumor volume has reached 120 ± 50 mm^3^, 3 groups received either injection of nanoparticles (AuNP) or irradiation (808nm; 10 min at 0.2 W/cm^2^). The group control 4 received an injection of Anti-PD1 monoclonal antibody. After the treatment, mice were weighed and the tumor volume was measured every 3 days until the death or sacrifice of the mice (mice were euthanized if the tumor volume was higher than 1500mm^3^ or if the body weight loss was higher than 20%).
Data source location	Institution: TORSKAL SAS City/Town/Region: Sainte Clotilde/La Reunion Country: France
Data accessibility	Data is available with the article, in supplementary material, and on a public repository (Mendeley data). The link is below: https://data.mendeley.com/submissions/ees/edit/kt4sww7h38?submission_id&amp;&equals;DIB_61012&amp;token&amp;&equals;aca6f526-4931-426f-88ad-21262ad6be5d
Related research article	Maroua Ben Hadadda, Dimitri Koshel, Zhang Yang, Weiling Fu, Jolanda Spadavecchia, Sabrina Pesnel, Anne-Laure Morel. Proof of concept of plasmonic thermal destruction of surface cancers by gold nanoparticles obtained by green chemistry. Colloids Surf B Biointerfaces. https://doi.org/10.1016/j.colsurfb.2019.110496

**Table:** 

**Value of the Data** • This research analyses the effect of gold nanoparticles coupled to infrared laser exposure on a murine melanoma model in order to determine the potential of plasmonic phototherapy as non-invasive alternative treatment to surgery. • In addition, the present article presents complementary information at lower power density (0.2W/cm^2^) and could be interesting for readers of the Data in Brief journal. A lower power density means less or no side effect on the skin due to the laser. • Such data can be used as a reference series for comparative approaches in plasmonic phototherapy requiring few damages to healthy tissues (no necrosis). This is useful for future studies on melanoma. • Comparison between the reference treatment (anti-PD1 monoclonal antibody) and plasmonic phototherapy.

## 1 Data description

Data shown in this article provide information about the effect of photothermal therapy on an aggressive murine melanoma model. Mice were subcutaneously engrafted with B16F10 murine melanoma cells in the right flank. The treatment started when the tumor volume reached 120 mm^3^ (Day 0). After the treatment, mice were weighed and the tumor volume was measured every 3 days until the death or the sacrifice of the mice (mice were euthanized if the tumor volume was higher than 1500 mm^3^ or if the body weight loss was higher than 20%). Tumor volume monitoring allows to determine the tumor growth in order to detect an inhibition or a regression of the tumor. The measurement of tumor volume with a digital calliper is the reference technique for subcutaneous models. Weight monitoring allows to determine the toxicity of the product; a product that induces a body weight loss greater than 20% can be considered as highly toxic.

Table 1 contains mice body weight (g) measured from the beginning (day 0 = D0) of the treatment to its sacrifice (day 10 = D10). These raw data were obtained from 25 melanoma bearing mice divided into 4 groups of mice:

- Group 1: injection of nanoparticles without laser
- Group 2: Laser exposure alone
- Group 3: Nanoparticles combined with laser
- Group 4: Reference treatment group (anti-PD1 monoclonal antibody).

**Table 1:** Body weight (g) in melanoma bearing mice from the treatment to their sacrifice.

Group	Mouse	Time (days)			
		D0	D3	D6	D10
Control	1–1	17.0	17.2	19.1	
	1–2	14.9	17.1	16.6	18.2
	1–3	16.6	14.7		
	1–4	16.4	16.1	17.2	18.3
	1–5	17.1	17.6	18.7	21.1
Laser	2–1	17.9	16.8	16.7	17.9
	2–2	18.1	15.8	16.8	19.5
	2–3	16.5	19.0	21.2	23
	2–4	16.7	13.8	18.7	19.2
	2–5	14.2	16.2	14.7	14.7
NP	3–1	13.7	14.7	15.2	15.5
	3–2	15.1	14.6	16.7	16.0
	3–3	16.8	18.6	17.1	
	3–4	16.7	18.0	19.8	24.2
	3–5	13.3	13.6	14.8	18.6
Anti-PD1 mAb	5–1	16.2	17.8	21.3	22.6
	5–2	17.6	17.6	20.1	20.2
	5–3	17.9	17.6	20.4	22.0
	5–4	17.7	19.6	18.7	20.3
	5–5	16.6	18.3	20.1	

Throughout this experimentation, mice were weighed, and their clinical state was rigorously controlled.

Table 2 describes the tumor mice dimensions of each group of mice (raw data). The tumors are measured from the beginning of the treatment (D0) to its sacrifice (D10). For each mouse, the width, the length and the thickness of the tumor were measured with an electronic calliper (expressed in mm). From these measurements the tumor volume was determined in order to follow the tumor growth.

**Table 2:** Tumor measurements (mm) obtained with a digital calliper from the treatment to their sacrifice.

Group	Mouse	Time (Days)/Tumor measurements^a^ (mm)											
		D0			D3			D6			D10		
		l	w	t	l	w	t	l	w	t	l	w	t
Control	1–1	9.6	6.6	4.1	14.5	10.0	7.6	18.7	15.1	10.2			
	1–2	9.3	7.8	4.8	13.4	8.4	6.1	16.8	12.1	8.6	21.8	17.8	8.7
	1–3	9.4	7.2	4.6	13.6	10.0	7.4						
	1–4	8.6	7.4	3.9	10.6	8.4	6.2	15.3	13.3	10.8	17.9	15.3	10.9
	1–5	7.1	7.0	3.8	11.8	9.4	5.9	14.3	13.4	9.2	19.0	15.1	10.0
Laser	2–1	9.6	6.7	4.0	12.2	8.8	5.9	13.5	11.7	9.4	18.8	14.8	10.2
	2–2	9.8	7.1	3.2	11.1	8.4	6.7	16.7	8.2	8.5	20.7	13.3	9.5
	2–3	9.4	6.8	3.6	12.4	11.3	7.5	15.8	12.0	10.8	20.9	16.0	8.9
	2–4	8.5	7.1	4.5	11.3	8.8	6.0	15.8	10.1	9.4	17.2	13.1	10.2
	2–5	8.2	7.7	4.1	13.8	8.5	6.6	16.3	13.0	8.7	19.4	17.5	6.9
NP	3–1	7.8	6.6	4.0	9.5	7.7	6.0	12.4	11.0	7.6	18.6	16.5	8.8
	3–2	7.9	6.4	4.2	8.3	7.7	3.3	12.1	8.4	5.5	20.8	16.0	8.2
	3–3	8.6	6.9	4.7	9.4	8.9	5.1	10.9	9.4	9.8			
	3–4	7.2	8.0	4.5	10.0	9.2	5.6	12.8	12.0	7.3	19.6	15.7	9.1
	3–5	8.8	7.0	4.1	7.9	6.9	4.6	11.3	10.4	6.4	17.8	16.6	8.3
Anti-PD1 mAb	5–1	8.5	8.2	3.5	12.1	10.7	8.0	13.2	11.4	8.9	18.0	15.6	9.0
	5–2	9.3	6.2	3.2	10.8	9.9	7.3	11.9	10.5	9.3	18.6	15.9	9.7
	5–3	10.2	7.3	3.5	14.5	13.7	7.0	15.2	13.8	8.2	19.5	15.2	9.7
	5–4	8.4	7.4	5.3	14.7	8.7	6.9	14.8	11.3	7.6	17.5	16.0	10.8
	5–5	9.3	7.6	4.6	13.0	10.7	6.6	16.1	15.2	8.5			

a l: length; w: width; t: thickness.

Table 3 presents the estimated tumor volume of each group of mice. The estimated tumor volume is calculated from the tumor mice measurements. This volume was calculated with the following mathematical formula: Volume = (width x length x thickness)/2, and was expressed in mm^3^. The tumor volume monitoring allows to determine the effect of the treatment on the tumor growth (no effect, inhibition or regression).

**Table 3:** Tumor volumes calculated from mice measurements^a^ (expressed in mm^3^).

Group	Mouse	Time (days)			
		D0	D3	D6	D10
Ctrl	1–1	128	550	1430	
	1–2	172	342	878	1679
	1–3	159	505		
	1–4	122	276	1102	1492
	1–5	93	328	873	1428
Laser	2–1	128	314	743	1415
	2–2	110	311	583	1302
	2–3	114	522	1032	1495
	2–4	136	300	745	1147
	2–5	129	384	921	1166
NP	3–1	102	219	520	1349
	3–2	105	105	277	1355
	3–3	138	212	503	
	3–4	129	255	562	1400
	3–5	127	126	376	1231
Anti-PD1 mAb	5–1	122	516	673	1251
	5–2	93	391	586	1442
	5–3	130	694	861	1435
	5–4	165	436	634	1503
	5–5	163	461	1037	

a Volume = (l∗w∗t)/2.

Table 4 contains the analysis of monitored weight and tumor volumes during the treatment from the groups of mice #3 and #4. This table summarizes the results obtained with the treatment on the body weight and on the tumor volume in order to determine if the treatment is toxic or not and if it has an antitumor activity or not. The relative area under the curve values were obtained from the median tumor volume curves. As an antitumor activity was shown in the related article no or low antitumor activity could mean that the power laser is too weak to be efficient with this dose of nanoparticles. Another study could be launched to test an intermediate power laser.

**Table 4:** Effects of PPTT against the subcutaneous B16F10 murine melanoma xenografts.

	Toxicity			Tumor growth	
	Maximal body weight change (%)^a^	Body weight change loss > 20% (%)	Presumed drug related deaths (%)^b^	rAUC (%)^c^	Optimal T/C (%)^d^
NP	−3.3	0	0	69	51
Anti-PD1 mAb	−1.7	0	0	89	68

a Body weight changes are gains or losses expressed as a percentage of the initial body weight. At each day of weighing, the median value of body weight changes is determined for each experimental group. Then the maximal body weight loss recorded over time is defined. According to NCI criteria, a dose is considered highly toxic if the induced body weight loss is greater than 20% of the initial body weight.

b A death is presumed to be drug-related if the treated animal died before the control animals. According to NCI, a dose is considered as toxic if the percentage of toxic deaths is higher than 20%.

c rAUC = relative area under the tumor growth curve (expressed as a percentage of the median area under the tumor growth curve of the control group).

d T/C = (median tumor volume of drug-treated group/median tumor volume of control group) x 100. The optimal T/C is the lowest value, reflecting the maximal tumor growth inhibition.

**Fig. 1:**
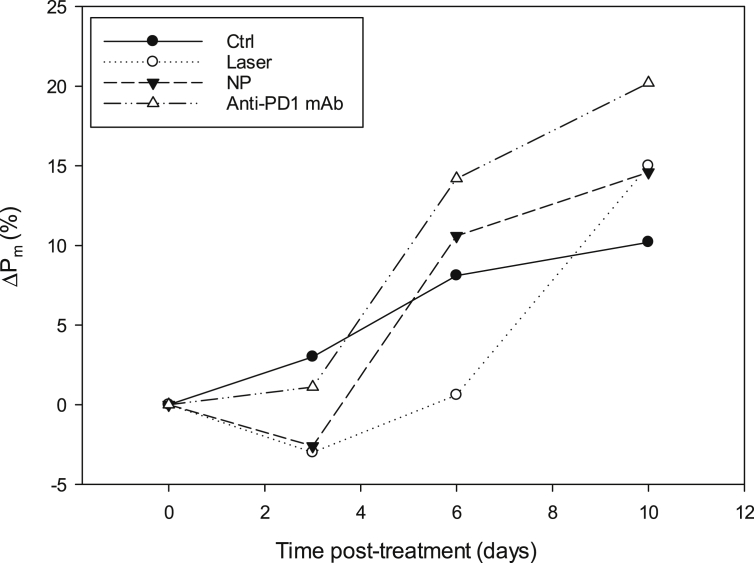
Evolution of body weight of mice bearing subcutaneous B16F10 murine melanoma xenograft.

Fig. 1 illustrates the evolution of the relative median body weight (%) of each group from D0 to D10.

No body weight loss was observed.

**Fig. 2:**
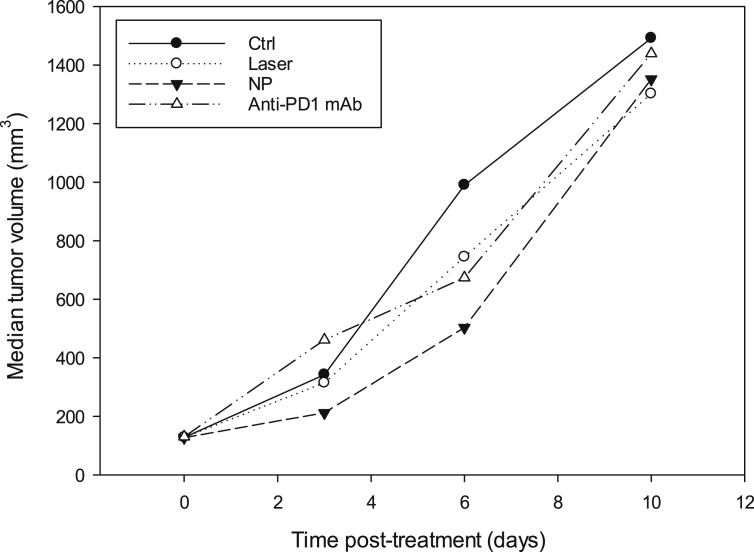
Evolution of tumor volume of mice bearing subcutaneous B16F10 murine melanoma xenograft.

Fig. 2 illustrates the evolution of the median tumor volume (mm^3^) of each group from D0 to D10.

## 2 Experimental design, materials, and methods

### 2.1 Nanoparticles (NP@G@P)

The gold nanoparticles were synthesized using the method previously described [2] then stabilized by a carbohydrate. These nanoparticles were then functionalized by a peptide (NP@G@P) as previously described [1]. The solution of nanoparticles was redispersed in PBS to get two different optical density: 0.6 and 1.2 (The UV–Vis absorption spectra was recorded by a PerkinElmer Lambda UV/Vis 950 using standard 1mm plastic cells at room temperature).

### 2.2 Origin of the tumor line – cell culture

B16F10 (ATCC® CRL-6475TM) is a murine melanoma cell line from a C57Bl/6J mouse. Cell line was cultured in RPMI 1640 medium supplemented with FBS (10%). Cells were maintained in an incubator at 37 °C in 5% CO_2_ and 95% air in a humidified atmosphere.

### 2.3 Animals and tumors

20 C57Bl/6 female mice, 6 weeks-old were used. Animals were housed in plastic cages inside a controlled ventilated rack with free access to water and food. All experiments were performed in accordance with animal national animal care guideline (EC directive 86/609CEE, French Decree n°87–848). Tumor xenografts were achieved by subcutaneous injection of tumor cells suspension (2.5 × 10^5^ B16F10 cells in 100μl PBS) into the right flank. To follow tumor growth, tumors were measured with a digital caliper and the volume was calculated as follow: Volume = (Width x Length x Thickness)/2.

Mice were weighed and tumors were measured every 3 days until the mice died or were sacrificed (sacrifice became required when the tumor volume have reached 1500 mm^3^ or the weight loss was upper than 20% of the initial weight).

### 2.4 Evaluation of toxic-side effects

Maximum weight losses or gains expressed as a percentage of the initial body weight of the experimental animals was used to provide an assessment of the toxicity of both AuNP. According to NCI (National Cancer Institute) criteria, a dose is considered toxic if the induced body weight loss is higher than 20% of the initial mouse body weight.

### 2.5 *In vivo* plasmonic photothermal therapy (PPTT)

When tumors reached a volume of 120 ± 50 mm^3^, mice were randomly allocated to 4 groups (5 mice per group) and the treatment started (Day 0) as follow: in the CTRL group, mice received an injection of NP@G@P (50 μL; OD = 1.2); in the laser group, PBS (50 μL) was injected into the tumor then the anesthetized mice received PPTT; in the NP group, NP@G@P (50 μL; OD = 0.6) was injected into the tumor then the anesthetized mice received PPTT. In the study design another NP group of mice was injected with NP@G@P (50 μL; OD = 1.2) (data not shown) it is why the control group was treated with the highest dose of NP. Tumor treatment was performed 4 hours after injection of PBS or NP@G@P. PPTT consisted in a tumor irradiation with an 808 nm laser diode during 10 minutes with a power density of 0.2 W/cm^2^. In the anti-PD1 mAb group, mice received an intraperitoneal injection of murine anti-PD1 mAb (5mg/kg) every 5 days according to the clinical treatment.

### 2.6 *In vivo* evaluation of PPTT efficiency

Treatment efficacy was assessed in terms of the compound's effects on tumor volume for PTT-treated mice relative to control vehicle-treated mice. Two evaluation criteria were used in parallel: (i) Growth inhibition, calculated as the ratio of the median tumor volume of NP-treated versus control groups: T/C, % = (median tumor volume of NP-treated group on day X/median tumor volume of control group on day X) x 100, the optimal value, being the minimal T/C ratio which reflects the maximal tumor growth inhibition achieved [3]; (ii) Relative area under the tumor growth curve, rAUC (%), representative of the tumor growth curve as a whole, reflects the overall effect of a test compound over time [4]. rAUC = [(area under the tumor volume growth curve of the treated group/median area under the tumor volume growth curve of the control group) x 100]. The more active the compound, the lower the rAUC value.

For further information concerning the experimental procedures please see the research article [1] accompanying this data paper.
